# Neonatal Hyperbilirubinemia treatment by Locally Made Low-Cost Phototherapy Units

**DOI:** 10.4314/ejhs.v31i1.7

**Published:** 2021-01

**Authors:** Netsanet Workneh Gidi, Matthias Siebeck

**Affiliations:** 1 Jimma University, Jimma, Ethiopia; CIHLMU Center for International Health, Medical Center of the University of Munich (LMU), Germany; 3 Medical Center of the University of Munich (LMU), Germany

## Abstract

**Background:**

Hyperbilirubinemia is a very common finding in neonates and may occasionally cause severe morbidity and even mortality. Severe hyperbilirubinemia is typically treated, either with phototherapy or exchange transfusions. This study assessed the effectiveness of a locally manufactured phototherapy device for reducing serum bilirubin in neonates with severe hyperbilirubinemia.

**Methods:**

Retrospective chart review was carried out to assess the outcome of 32 infants who were treated for neonatal hyperbilirubinemia at Jimma Medical Center (JMC) from May, 2017 to April, 2018. RESULTS: Out of 75 charts reviewed, only 32 had subsequent bilirubin level determination, 18(56.3%) of them were males. The age at which jaundice was noticed and confirmed with plasma bilirubin level was 4 ± 2.7 days (mean±SD). Sepsis was thought to be the cause of hyperbilirubinemia in 13(40.5%) of the cases, while hemolysis from ABO incompatibility or RH incompatibility contributed in 5(15.6%) and 3(9.4) of the infants respectively. The mean (minimum, maximum) level of baseline TSB was 21.4(14, 55) mg/dL. Five infants (15.6%) had exchange transfusions because of extreme hyperbilirubinemia. The duration of phototherapy and decline in TSB were 5.34 ±2.8 days and 2.2±1.5mg/dl/day (mean±SD) respectively. The levels of TSB before and at the end of phototherapy were significantly different (p<0.001).

**Conclusion:**

Acceptable reduction of TSB was achieved by using locally manufactured PT devices. Benefits included better accessibility and lower price and maintenance costs. High mean baseline TSB was observed, and duration of phototherapy is prolonged which could indicate late diagnosis compared to similar studies.

## Introduction

Acute bilirubin encephalopathy (ABE) or kernicterus is a feared complication arising from untreated severe neonatal hyperbilirubinemia and a leading cause of preventable brain damage (1,2). This unacceptably high global burden of severe neonatal hyperbilirubinemia and associated brain damage is a preventable and/or treatable disorder that requires implementation of existing technology to reduce the associated morbidity and mortality (3). Neonatal hyperbilirubinemia is a very common and mostly benign transitional condition that can be seen during the first few days of life in 60%–80% of newborns worldwide (4). Further studies are highly needed to understand the profile of infants with or at risk of severe hyperbilirubinemia in most low-income and middle-income countries (LMICs) (5).

Although the term “severe hyperbilirubinemia” is not strictly defined, the following elements have been proposed: a newborn who develops visible jaundice within the first 24 hours of life, when total serum bilirubin (TSB) levels increase >5 mg/dl/day, when peak levels are higher than the expected normal range (i.e.>95^th^ percentile), when clinically visible jaundice persists for more than 2 weeks after birth, and when there is evidence of conjugated hyperbilirubinemia (6). Kernicterus Spectrum Disorder (KSD) is characterized by long-term neurodevelopmental abnormalities, including dyskinetic (choreoathetoid) cerebral palsy, paresis of upward gaze, developmental delay, cognitive impairment, disordered executive function, language processing disorders, as well as behavioral and psychiatric disorders (7).

Common causes that can contribute to severe neonatal jaundice (NJ) include hemolysis due to Rh factor or ABO blood group incompatibility, glucose-6-phosphate dehydrogenase (G6PD) deficiency and other hemolytic disorders, as well as breastfeeding or breast milk, premature birth, infection, cephalohematoma or other circumscribed hemorrhages, and asphyxia (8,9). Timely identification of risk factors that predispose to severe hyperbilirubinemia and neurotoxicity is important in order to be able to start treatment on time and thus decrease mortality and morbidity (3).

Phototherapy is the preferred method of treatment for severe neonatal unconjugated hyperbilirubinemia regardless of its etiology. Phototherapy works most effectively at blue light wavelengths (450 ± 20 nm), preferably placed close to the infant (30 cm distance or less), and with as much of the infant's body surface area exposed as possible, in order to maximize spectral power (10,11). Guidelines for phototherapy exist in several formats and typically relate TSB thresholds to the infant's maturity (either represented by gestational age or birthweight), postnatal age (in hours), and presence of factors/conditions that increase the risk for bilirubin neurotoxicity(12). Such risk factors may include isoimmune hemolytic disease, glucose-6-phosphate dehydrogenase deficiency, asphyxia, lethargy, temperature instability, sepsis, acidosis, and hypoalbuminemia (12,16). Exchange transfusion (ET) is considered the most effective measure to rapidly lower the bilirubin level to prevent acute bilirubin encephalopathy and progression to KSD. However, unlike phototherapy, it is invasive and associated with complications of blood transfusion and the procedure (3,13–15).

The problem of severe NJ and gaps in implementing effective strategies for identification, prevention, and treatment are still significant in several LMICs. Standard phototherapy machines are expensive and not readily available in the market in many LMICs. Thus, there is a great demand for affordable alternative devices in these settings (4,19,20). The aim of this study was to assess how well locally made low cost phototherapy devices worked in the treatment of NJ.

## Materials and Methods

The study was conducted in the neonatal intensive care unit (NICU) at JMC, Jimma, Ethiopia. JMC is one of the tertiary hospitals in Ethiopia, located in the Southwest part of the country, with over 600 beds capacity. The pediatric unit of JMC has a NICU with over 25 beds. Neonates admitted to the unit are either referred from the surrounding health centers or the JMC delivery room. JMC NICU has been using locally made phototherapy units since May 2017, as there was no functional phototherapy unit available at the hospital. Several expensive machines were out of use at the same time, due to lack of maintenance and lack of availability of spare parts.

This was a retrospective cross-sectional study. Neonates (age <28 days) admitted with the diagnosis of hyperbilirubinemia from May 2017 to April 2018 at Jimma Medical Center were the study population. Neonates diagnosed with hyperbilirubinemia were identified from neonatal ward logbook, and patient charts were retrieved. The data was collected using structured chart review guide developed for this study. Data was collected by physicians from neonatal ward logbook and patient charts using structured chart review guide. The data collectors were trained for the purpose of the study and study procedures prior to commencement of data collection. Patient and mothers' characteristics, ABO blood group and RhD antigen typing of mother and child, other possible causes of hyperbilirubinemia, levels of serum bilirubin, duration of therapy, and outcome were reviewed using a structured chart review guide. Data was checked for completeness, cleaned, and entered into SPSS version 22 for analysis. Descriptive statistics was used where appropriate. Paired sample T test was done to assess for statistically significant reduction of TSB after treatment with phototherapy. The study was conducted after ethical approval was obtained from Jimma University IRB, reference number: IHRPGD/456/2018.

Simbona Africa Healthcare R & D, an organization founded by Mr. Habtamu Abafogi, provided the locally made phototherapy units which the NICU has been using for about a year. The components used to construct phototherapy units comply with medically recommended specifications (Table 1). The machine is easy to install and maintain, lamps can be replaced easily, for LED's the whole board can be replaced by technicians on site, it can be disinfected by available disinfectants and can operate at 25 °C -50 °C. Technical (engineering) evaluation of the phototherapy units was done by Jimma University Biomedical Engineering Centre. Irradiance level was 18µW/cm2/nm as measured by a radiometer. Electronics board safety, wavelength, and irradiance level were confirmed to be in accordance with international standard guidelines ISO 900:IEC 60601-52 for neonatal phototherapy units (reference number: BME-JUMC-182-10).

**Table 1:** Specifications of basic technology used to construct phototherapy units by Simbona Africa Healthcare R&D, Ethiopia

No	Components	Specifications	Brand	Companies currently using the components
**1**	Blue LED	- Wave length (450–470nm) - SMD Blue LEDs - DIP Blue LEDs - Life time:50000hrs	CE-Market	- Fanem Phototherapy - Natus PT
**2**	CFL	- Wavelength 450nm - 10,000hours	CE-Market	- Olympic - Sylvania - neoBlue - Philips and other
**3**	Timer	6 digit counter	CE-Market	Philips phototherapy

## Results

**Figure 1:**
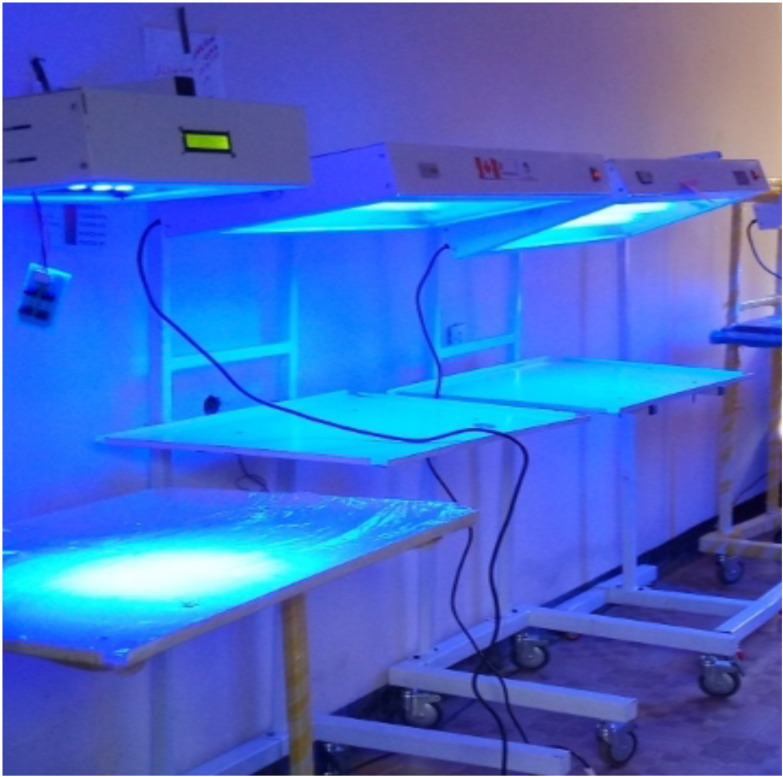
Phototherapy devices developed by Simbona Africa Healthcare R&D, Ethiopia.

Four locally made phototherapy units were in use: two CFL and two LED. There were 87 infants diagnosed with neonatal jaundice requiring treatment, accounting for 8.5% of total NICU admission in the year. Out of 75 charts retrieved (12 charts could not be accessed), only 32 had follow-up TSB determinations. The rest were either treated on clinical basis without measuring TSB or did not have hyperbilirubinemia in the range phototherapy is indicated (Figure 1). The report thus covers the outcome of 32 infants who had adequate TSB determinations both before and after treatment with phototherapy.

The majority of infants 19(59.4%) were born in JUMC; while 11 (34.4%) were referred from the surrounding health centers and hospitals, the remaining 2(6.3%) were born at home. All of the mothers had antenatal care, of these 4 were identified with antenatal hemorrhage, preeclampsia, Rh- negative mother, and thyrotoxicosis. The mode of delivery was spontaneous vertex delivery in 27 (62.8%) of the cases, while 10(31.3%) and 2(6.3%) were born by caesarian section and instrumental delivery, respectively.

Eighteen (56.3%) infants were male. The age at which jaundice was clinically noticed and confirmed with a TSB level was day 4 ± 2.7 (mean ± SD). As routine screening is not included in the guidelines for the unit, all the infants were tested following clinical suspicion of severe jaundice. The birth weight and GA of the infants were 2825 ± 817G and 36.8 ± 2.6 weeks respectively (mean ± SD). Sepsis was identified as the main cause of hyperbilirubinemia in 13(40.5%) of the cases, while hemolysis from ABO incompatibility and Rh incompatibility were seen in 5(15.6%) of the infants. Only one of the infants had clinical signs of acute bilirubin encephalopathy on admission. The level of TSB and HCT were 21.4 (14, 55) and 44.7(28, 62) respectively (mean (range) (Table 2).

**Table 2:** Perinatal data of infants with pathologic jaundice

Variables	Values
Males No. (%)	18(56.3)
GA weeks, mean (SD)	36.8(2.6)
Birth weight G, mean (SD)	2825(817)
Parity of the mother, mean (SD)	2.7(1.8)
Place of delivery, No. (%)	
JUMC	19(59.4)
Health centers and other hospitals	11(34.4)
Home	2(6.3)
Mode of delivery, n(%)	
Spontaneous vaginal	20(62.5)
Caesarean section	10(31.3)
Instrumental	2(6.3)
Age at diagnosis of hyperbilirubinemia in days, mean (SD)	4(2.7)
Causes of hyperbilirubinemia No. (%)	
Sepsis	13(40.5)
ABO incompatibility	5(15.6)
Rn incompatibility	3(9.4)
Subgaleal hemorrhage	3(9.4)
Breast feeding jaundice	2(6.3)
Hypothyroidism	1(3.1)
Unknown cause	5(15.6)
TSB (g/dL) at start of phototherapy, mean (range)	21.4(14,55)
Hematocrit, mean (range)	44.7(28,62)

Five of the babies had TSB value above the level at which exchange transfusion is indicated. However, only three of them got exchange transfusion while the other two were treated with phototherapy. The total duration of phototherapy and average decline in TSB level were 5.34±2.8 days and 2.2±1.5 mg/dl/day (mean±SD). The mean reduction of TSB after exchange transfusion was 9.9mg/dL/procedure. Paired sample test analysis showed significant difference in average levels of TSB before and at after phototherapy (t_31_=6.2, p <0.001). The majority of infants, 31(96.9%), were discharged with improved condition while one of the infant's family left against medical advice (Table 3).

**Table 3:** Treatment and outcome of hyperbilirubinemia (No =32)

Variables	Values
TSB>Exchange transfusion level, No. (%)	5(15.6)
Total duration of phototherapy in days, mean (SD)	5.34 (2.8)
Average decline in TSB in mg/dl/day, mean (SD)	2.2(1.5)
Average decline in TSB in mg/dl/procedure, mean (SD)	9.9(3.3)
Outcome at discharge	
Survived	31(96.9)
Left against medical advice	1(3.1)
Death	0(0)

## Discussion

Herein we show that locally made phototherapy units can be used effectively to treat neonatal hyperbilirubinemia. The lamps used in our devices were sourced locally, a significant advantage compared to imported units, for which spare parts and lamps may either not be available at all, or at significantly higher cost or long delays as far as delivery from the manufacturers (10). Although the basic concept and design of phototherapy devices is rather simple, technological sophistication in the newer generations of such devices means that the standard models on the market are too expensive for resource poor settings, with costs ranging from US$1200 to 1500, the manufacturing cost of the locally made phototherapy was about 400 US$. Many devices currently in use in LMIC hospitals were donated from hospitals in developed countries, often not accompanied by adequate supplies of spare parts, and they are not often checked for proper functioning, including irradiance (21,26). Because severe NJ continues to be an important health problem in LMICs (5), with the tragic sequelae of KSD contributing significantly to the burden of neurodevelopmental impairment in LMIC populations (4); many ways to increase the availability of treatment tools deserve to be explored (10,14).

A variety of phototherapy devices are considered effective in decreasing TSB (21). The mean daily TSB decrement observed in this study (2.1mg/dl) is comparable with the finding of others. Thus, Brown et al used daylight fluorescent tubes and reported a TSB decrease of 1.5 mg/dL during the first day of phototherapy (22). However, our numbers are lower than those in a report of a study that compared the effectiveness of LED versus CFL, with average decreases of TSB levels of 4.1 ± 0.48 and 4.8 ± 0.24 mg/dL respectively at the end of 24 hours (23).

The mean duration of phototherapy of 5.3 days in our study is longer than the findings of similar studies, average of 1–3 days which can be explained by late diagnosis requiring prolonged period to treat, lack of universal screening of newborns with TSB or cutaneous bilirubin, TSB being determined only when jaundice is clinically diagnosed. On the same line, the mean baseline TSB of the infants was greater than the levels reported by studies compared (24,25). Generally, phototherapy is considered safe with minimal risk, and none of the infants treated with PT in this study had reports of significant complications (24).

Measuring the dose of phototherapy is required to insure the efficacy of the device (12). However, determining device irradiance is not widely practiced, both due to lack of irradiance meters (radiometers specific to the device as recommended by manufacturer) (12), and also to limited knowledge of its importance by health professionals. Lack of maintenance and unavailability of replacement parts are also major impediments to sustainable, effective PT units in LMICs (21, 26). The manufacturing cost of the present locally made CFL and LED phototherapy units is about one-fourth of the price of the standard models in the market, with the possibility of maintenance at the site in case of malfunctioning and irradiance checks, which makes it attractive given the high demand.

The limitation of this study include retrospective data collection, and associated incomplete patient data. This forced us to exclude some neonates who had neonatal jaundice and received phototherapy. Some of the cases did not have adequate follow up bilirubin measurement recorded on their charts. For these reasons smaller number of neonates receiving phototherapy were included for analyis.

Acceptable decrement in TSB was achieved by using locally made phototherapy devices (CFL and LED). Additional benefits include better accessibility, lower cost and easier access to maintenance. We observed a high mean baseline TSB, and duration of phototherapy was prolonged, pointing to late diagnosis compared to similar studies. The cause of prolonged duration of therapy could be related to the fact that the phototherapy was conventional instead of intensive. However, understanding the exact reason why duration of therapy was prolonged requires further investigation. Adopting universal screening for hyperbilirubinemia with TSB or noninvasive cutaneous bilirubin determination could help to identify infants in need of therapy earlier.
